# Identification of key genes in ruptured atherosclerotic plaques by weighted gene correlation network analysis

**DOI:** 10.1038/s41598-020-67114-2

**Published:** 2020-07-02

**Authors:** Bao-Feng Xu, Rui Liu, Chun-Xia Huang, Bin-Sheng He, Guang-Yi Li, Hong-Shuo Sun, Zhong-Ping Feng, Mei-Hua Bao

**Affiliations:** 1 Department of Neurosurgery, the First Hospital of Jilin University, Changchun, Jilin, 130021 China; 2 Department of VIP Unit, China-Japan Union Hospital of Jilin University, Changchun, 130033 China; 3 Science Research Center, Changsha Medical University, Changsha, 410219 China; 4 Academician Workstation, Changsha Medical University, Changsha, 410219 China; 5 Department of Surgery, Faculty of Medicine, University of Toronto, Toronto, ON Canada; 6 Department of Physiology, Faculty of Medicine, University of Toronto, Toronto, ON Canada

**Keywords:** Computational biology and bioinformatics, Genetics, Molecular biology, Biomarkers, Diseases

## Abstract

The rupture of atherosclerotic plaques is essential for cardiovascular and cerebrovascular events. Identification of the key genes related to plaque rupture is an important approach to predict the status of plaque and to prevent the clinical events. In the present study, we downloaded two expression profiles related to the rupture of atherosclerotic plaques (GSE41571 and GSE120521) from GEO database. 11 samples in GSE41571 were used to identify the differentially expressed genes (DEGs) and to construct the weighted gene correlation network analysis (WGCNA) by R software. The gene oncology (GO) and Kyoto Encyclopedia of Genes and Genomes (KEGG) enrichment tool in DAVID website, and the Protein-protein interactions in STRING website were used to predict the functions and mechanisms of genes. Furthermore, we mapped the hub genes extracted from WGCNA to DEGs, and constructed a sub-network using Cytoscape 3.7.2. The key genes were identified by the molecular complex detection (MCODE) in Cytoscape. Further validation was conducted using dataset GSE120521 and human carotid endarterectomy (CEA) plaques. Results: In our study, 868 DEGs were identified in GSE41571. Six modules with 236 hub genes were identified through WGCNA analysis. Among these six modules, blue and brown modules were of the highest correlations with ruptured plaques (with a correlation of 0.82 and −0.9 respectively). 72 hub genes were identified from blue and brown modules. These 72 genes were the most likely ones being related to cell adhesion, extracellular matrix organization, cell growth, cell migration, leukocyte migration, PI_3_K-Akt signaling, focal adhesion, and ECM-receptor interaction. Among the 72 hub genes, 45 were mapped to the DEGs (logFC > 1.0, p-value < 0.05). The sub-network of these 45 hub genes and MCODE analysis indicated 3 clusters (13 genes) as key genes. They were LOXL1, FBLN5, FMOD, ELN, EFEMP1 in cluster 1, RILP, HLA-DRA, HLA-DMB, HLA-DMA in cluster 2, and SFRP4, FZD6, DKK3 in cluster 3. Further expression detection indicated EFEMP1, BGN, ELN, FMOD, DKK3, FBLN5, FZD6, HLA-DRA, HLA-DMB, HLA-DMA, and RILP might have potential diagnostic value.

## Introduction

Atherosclerosis is a chronic inflammatory disease responsible for many clinical manifestations, such as coronary artery disease and ischemic stroke^1^. The deposition of lipid under a blood vessel wall leads to the formation of atherosclerotic plaques. The rupture of the plaque blocks the blood vessel and is the main reason for cardiovascular or cerebral vascular events^2^. Identification of the key genes related to plaque rupture is an important approach to predict the status of plaque and to prevent the clinical events.

With the development of new technologies, microarray analysis has been a vital method for the research of genetic alteration in various diseases^3^. To deal with the big data from microarray profile, bioinformatics analysis has become a useful tool. For instance, linear models for microarray data (LIMMA) was used to identify the differential expressed genes (DEGs) from big data, Gene Oncology (GO) and Kyoto Encyclopedia of Genes and Genomes (KEGG) were used to predict the functions and mechanisms of genes^4^. Weighted gene correlation network analysis (WGCNA) is an advanced data mining method. It is used to describe the correlation patterns among genes, to find the clusters (modules) of highly correlated genes, to identify the hub genes in each module, and to associate the modules to external sample traits^5^. WGCNA provides another powerful method to group the genes into modules and coordinate the modules to biological functions and regulatory mechanisms^6^. WGCNA has been widely used in cancers, cardiovascular diseases and many other diseases^7,8^.

Recently, some microarray studies have been conducted to compare the gene expression profiles between carotid atheroma and the microscopically intact tissue (GSE43292); human normal arterial intimae and advanced atherosclerotic plaques (GSE97210); and ruptured and stable atherosclerotic human plaques (GSE41571, GSE120521)^9,10^. WGCNA was used to identify the key lncRNAs and mRNAs associated with atherosclerosis or coronary artery disease progression^8,11^. The bioinformatics method LIMMA was used to identify key genes in GSE41571. COL3A1, COL1A2, ASPN, PPBP were thought to play critical roles in atherosclerotic plaque rupture^10^. However, they only identified individual DEGs in microarray profiles. No WGCNA analysis was conducted on stable and ruptured atherosclerotic plaques.

In the present study, we downloaded the datasets GSE41571 and GSE120521 from GEO database (https://www.ncbi.nlm.nih.gov/geo/), identified key genes by using bioinformatics tools, such as LIMMA, WGCNA, GO, KEGG, and Cytoscape. The key genes were further validated in human carotid endarterectomy (CEA) plaques.

## Results

### Datasets and workflow

The datasets GSE41571 and GSE120521 were downloaded from the GEO database (http://www.ncbi.nlm.nih.gov/geo/). GSE41571 contains 5 ruptured and 6 stable atherosclerotic plaques, while GSE120521 contains 4 ruptured and 4 stable plaques.

**Figure 1:**
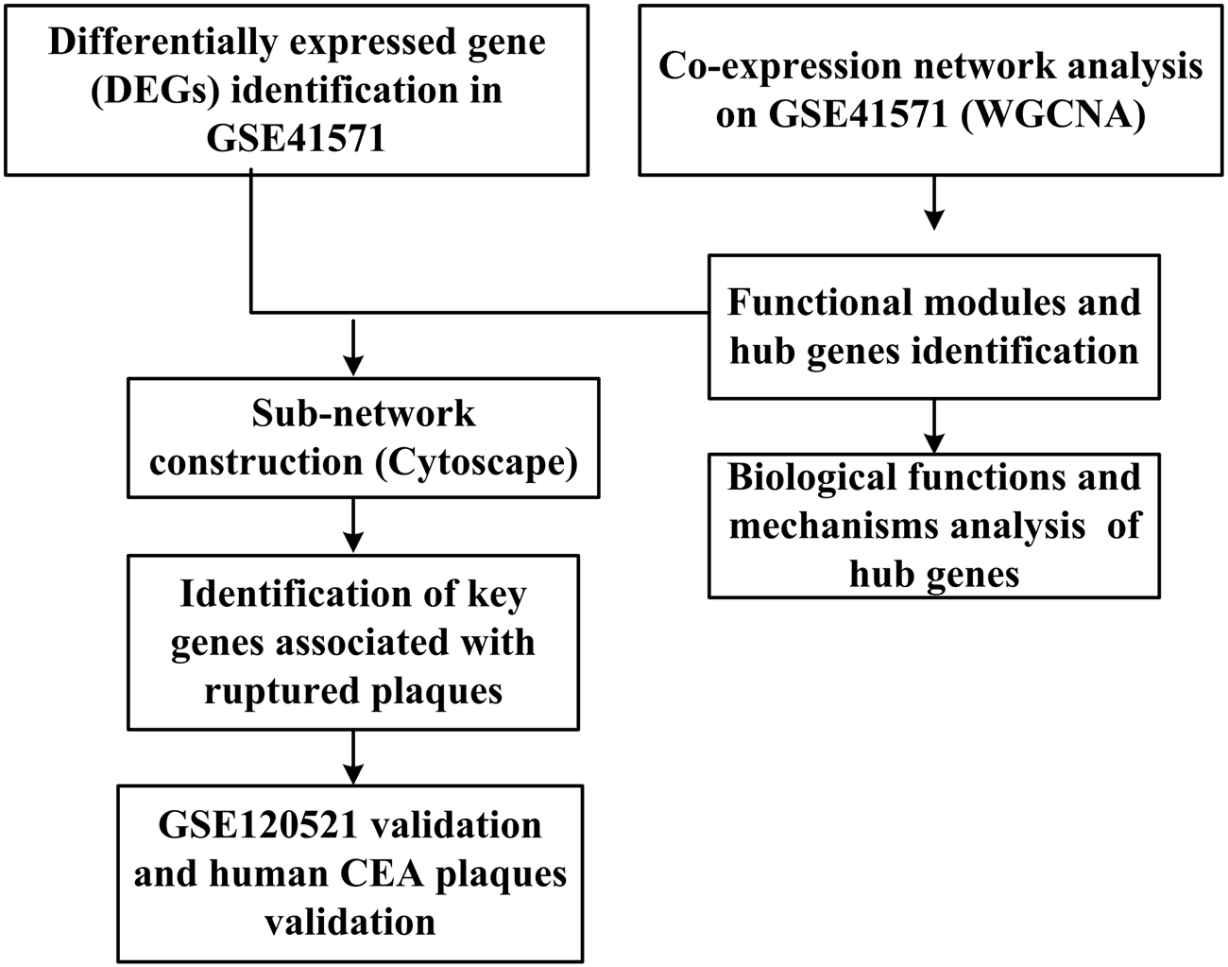
Workflow of the whole study.

The workflow of the whole study was shown in Fig. 1. DEGs in GSE41571 were firstly screened. The WGCNA was conducted on the ruptured and stable plaques in GSE41571. The functional modules and the hub genes were identified. Then the hub genes were mapped to the DEGs and formed a sub-network by Cytoscape. Key genes were identified based on this network. The key genes were validated using GSE120521 and human CEA plaques.

### Identification of the DEGs

**Figure 2:**
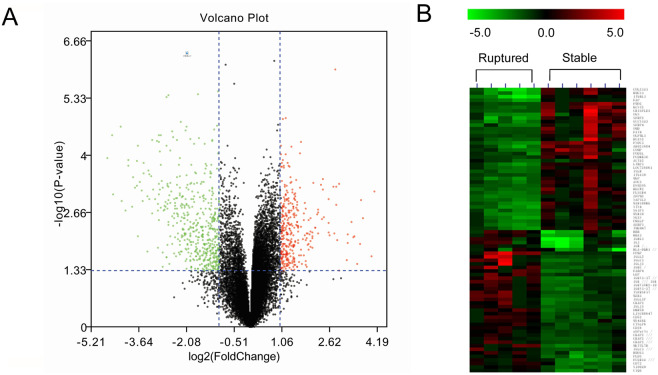
Identification of DEGs in GSE41571. A: volcano plot of GSE41571; B: Heatmap of the top 40 upregulated and top 40 downregulated DEGs.

The matrix file from GSE41571 was pre-processed and annotated with the official gene symbol. The DEGs were identified using “LIMMA” package in the R software^12^. The genes with fold change over 2.0 (logFC > 1.0) and *p*-value < 0.05 were considered DEGs. Eventually, 868 genes were identified (Fig. 2A). Compared to the stable plaques, 342 genes were upregulated and 526 genes were downregulated in the ruptured plaques. The heatmap of the top 40 upregulated and 40 downregulated DEGs were shown in Fig. 2B, and Table S1.

### Construction of gene co-expression networks by WGCNA

The gene expression profile GSE41571 (11 samples, 8413 genes) was downloaded. The top 50% variant genes (4207 genes) in GSE41571 were screened out by LIMMA and were used for the construction of gene co-expression network by WGNCA in the R software.

**Figure 3:**
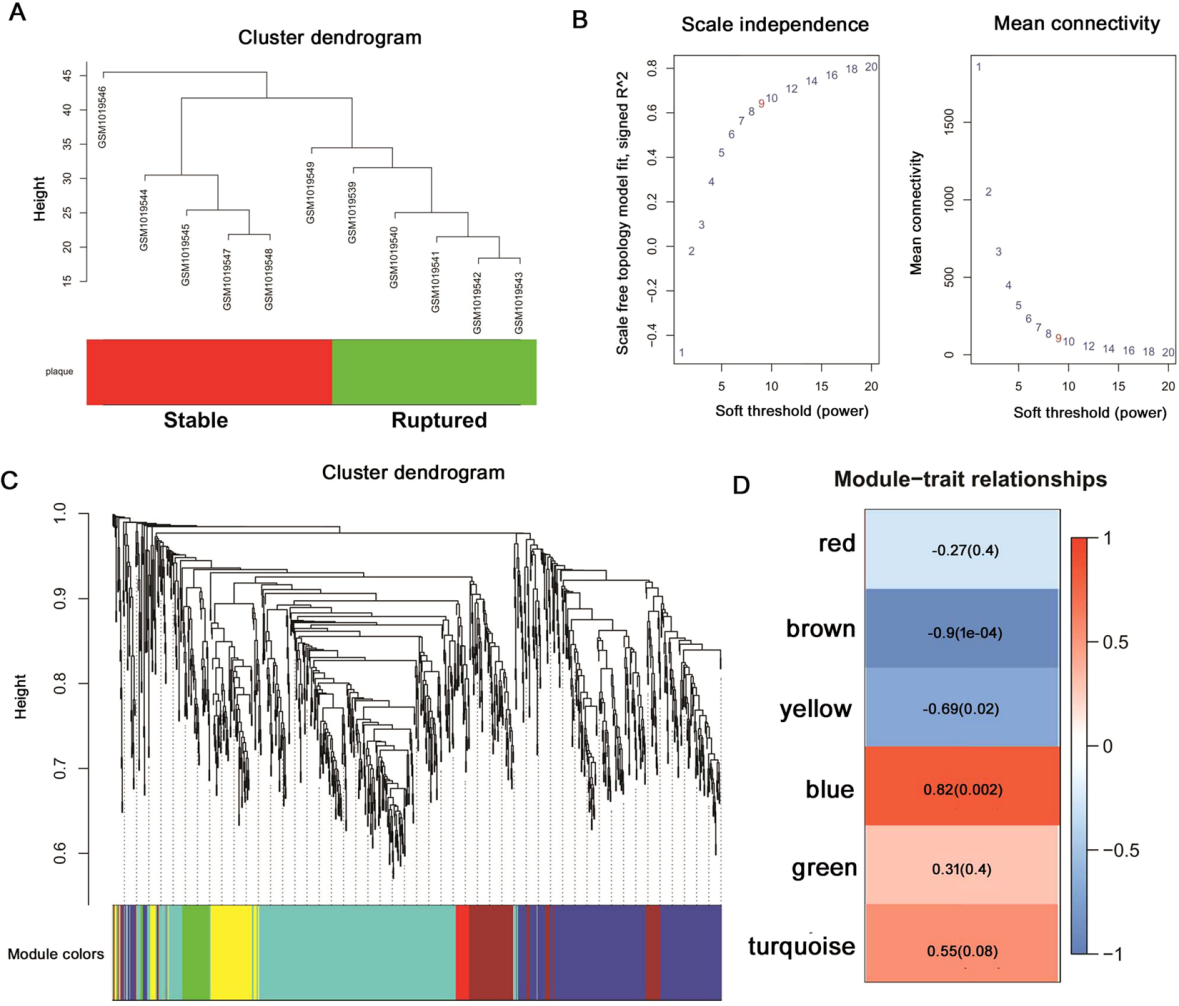
WGCNA identification of plaque rupture related hub genes. (**A**) Clustering dendrogram of 11 samples; (**B**) Determination of soft-threshoding power in the WGCNA; (**C**) Dendrogram of all differentially expressed genes clustered based on a dissimilarity measure (1-TOM); (**D**) Association between each individual module and the rupture of atherosclerotic plaque;.

The plaque traits of each sample were shown in Fig. 3A. To ensure a scale-free network for the construction of the co-expression network, a power of β = 9 was selected as a soft-thresholding parameter (Fig. 3B). The hierarchical clustering based on the topological overlap matrix (TOM) dissimilarity measure revealed six modules, namely, the blue, brown, green, red, turquoise, and yellow modules (Fig. 3C). Each module contained a group of coordinately expressed genes with high TOM value and was potentially related to the same clinical traits. Genes with the absolute values of Pearson’s correlation over 0.9 were considered hub genes. The number of genes involved in each module was 319 for the blue module (48 hub genes), 124 for the brown module (24 hub genes), 51 for the green module (15 hub genes), 22 for the red module (14 hub genes), 391 for the turquoise module (103 hub genes) and 88 for the yellow module (32 hub genes) (Table S2). Among these modules, blue and brown modules were found to have the highest association with atherosclerotic plaque rupture (correlations of 0.82, *p*-value of 0.002 for the blue module, and correlations of −0.9, *p*-value of 0.0001 for the brown module) (Fig. 3 D). Therefore, the hub genes in blue and brown modules were used for further analysis.

### Gene Oncology (GO) and Kyoto Encyclopedia of Genes and Genomes (KEGG) enrichment of hub genes in blue and brown modules

To analyze the functions and mechaisms of the identified hub genes in blue and brown modules, GO and KEGG enrichment in DAVID (website: https://david.ncifcrf.gov/) were used. The q-value < 0.05 was considered to be statistically significant for the correlations. The top 10 GO enrichment terms were visualized in the bubble charts by the Omicshare online tool (http://www.omicshare.com/tools).

**Figure 4:**
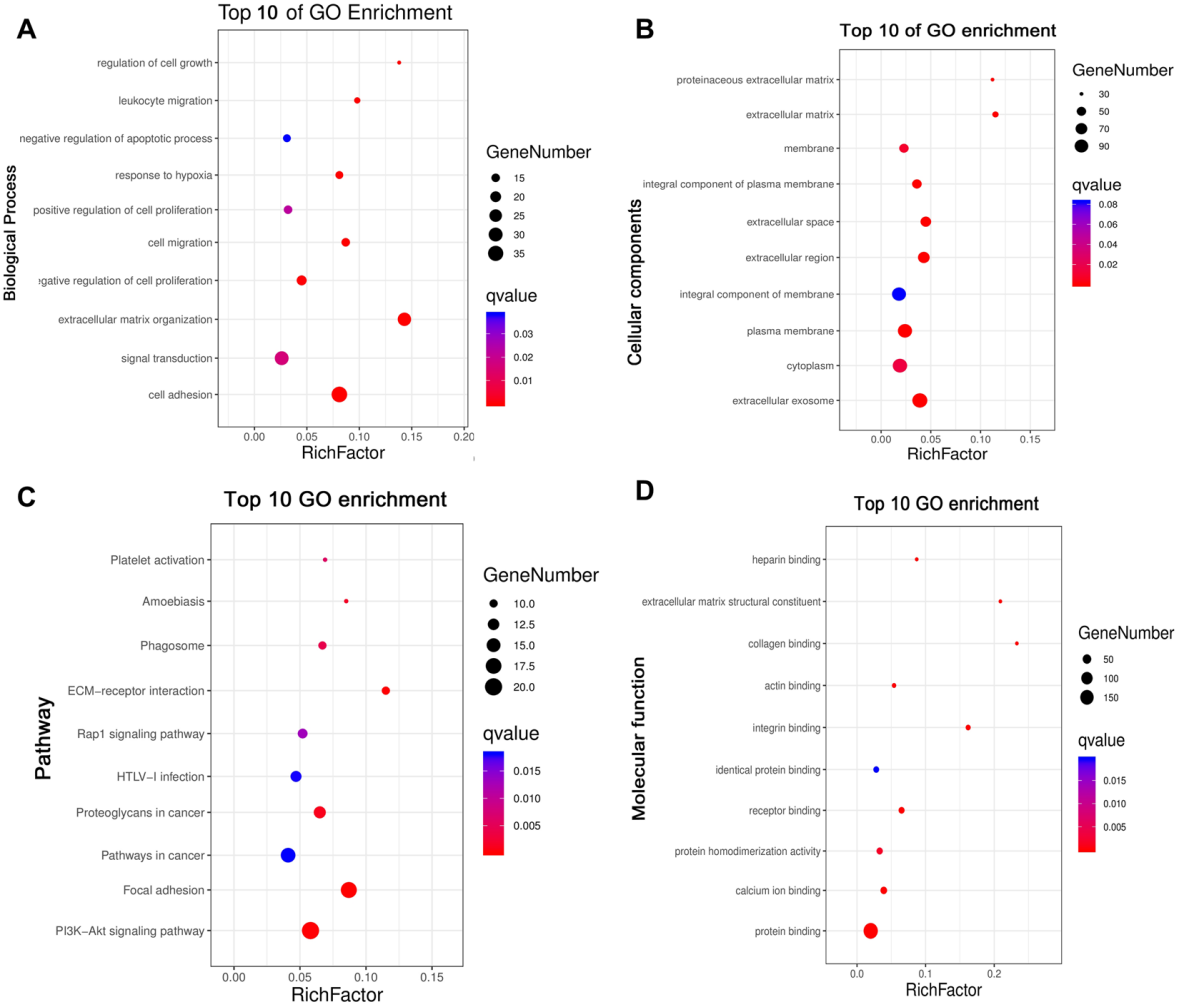
GO and KEGG enrichment of genes in blue and brown modules. (**A**) Biological Process of GO enrichment; (**B**) Cellular components of GO enrichment; (**C**) KEGG pathway enrichment; (**D**) Molecular function of GO enrichment; RichFactor = “count”/ “pop hits”. The “counts” is the number of hub genes enriched in a certain term. The “pop hits” is the number of all genes enriched in a certain term.

As shown in Fig. 4, these hub genes were mostly enriched in Biological Process (BP) of cell adhesion and extracellular matrix organization (Fig. 4A); Cellular Components (CC) of extracellular exosome, plasma membrane (Fig. 4B); and Molecular Functions (MF) of protein binding (Fig. 4D). KEGG enrichment indicated the genes most likely affect the signal pathways of PI3K-Akt signaling and focal adhesion (Fig. 4C).

### Protein-protein interaction (PPI) network of the hub genes in the blue and brown modules

**Figure 5:**
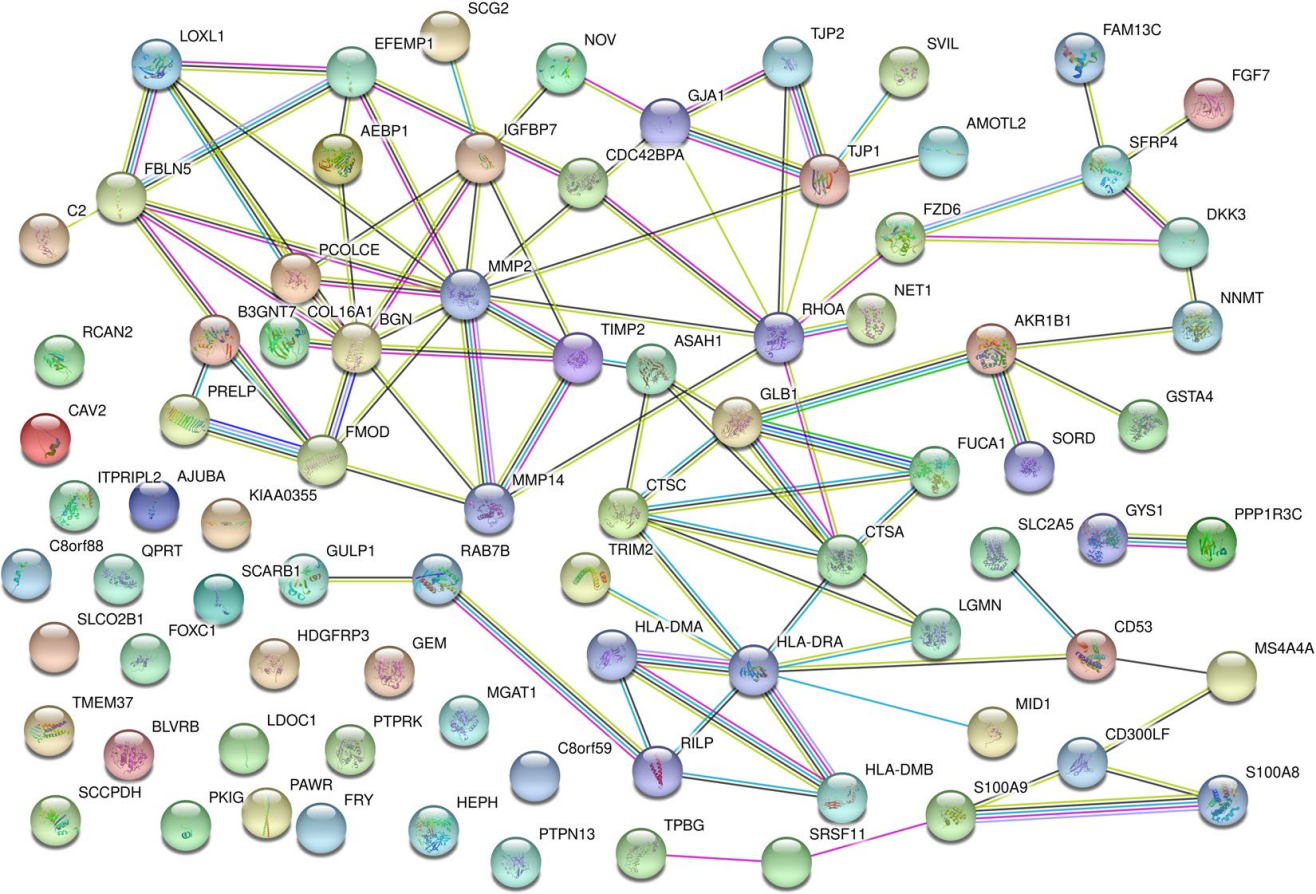
PPI network for the 72 hub genes in blue and brown modules. The circles represent the proteins encoded by the corresponding genes; lines represent the interactions between the proteins.

The PPI was analyzed by the STRING online tool (website: https://string-db.org/). 72 hub genes in the blue and brown modules were analyzed. As a result, the 72 hub genes formed a network with 82 nodes and 97 edges (Fig. 5). BGN, CTSA, FBLN5, and HLA-DRA were at the core of the network. These genes are mainly involved in the process of collagen formation and extracellular matrix degradation, and the functions of the immune system. All of the above pathological processes play critical roles in atherosclerosis.

### Identification of key genes

**Figure 6:**
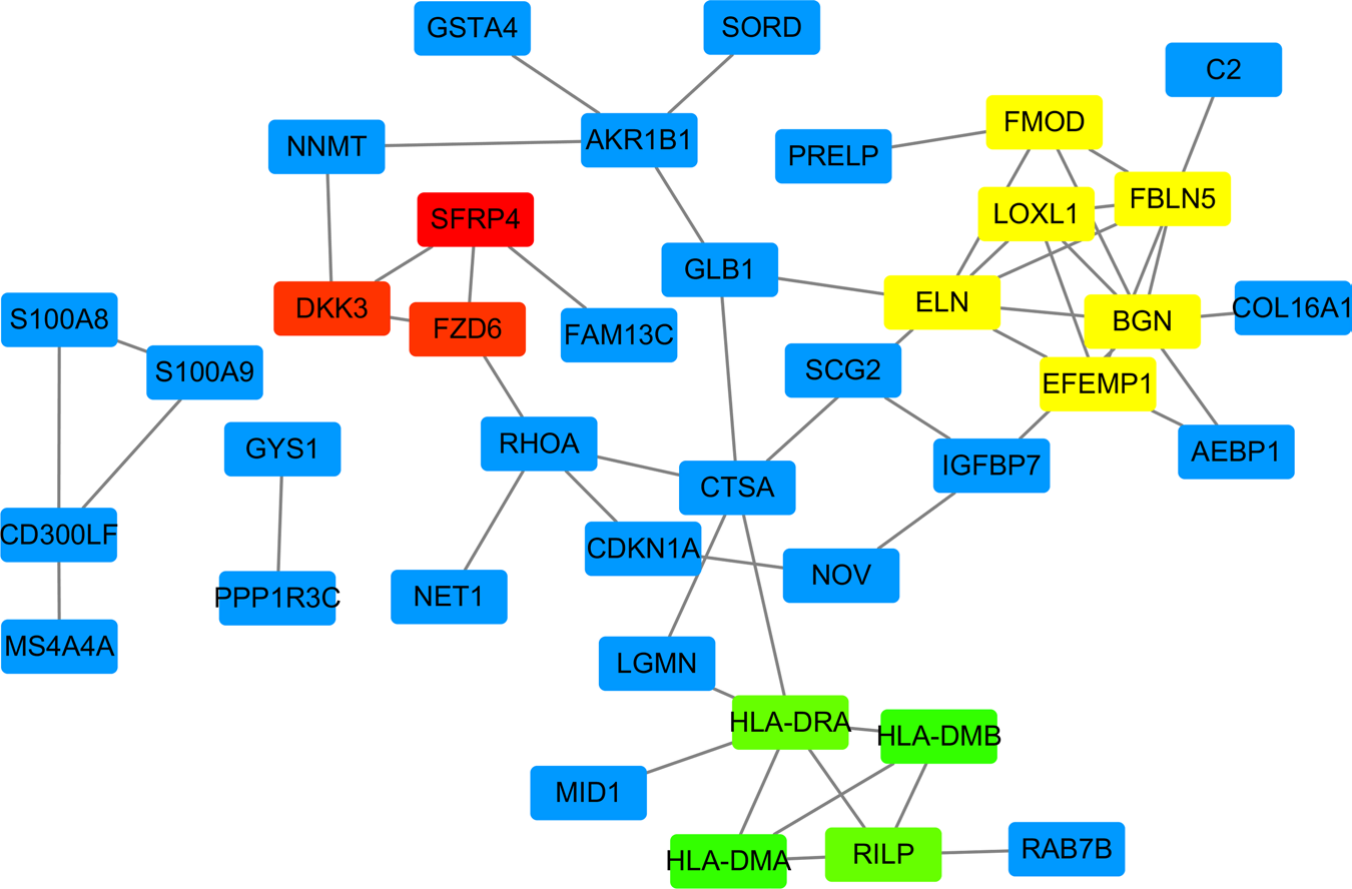
The 45 hub genes sub-network constructed in Cytoscape 3.7.2 software. Yellow boxes were key genes in cluster 1; green boxes were key genes in cluster 2; red boxes were key genes in cluster 3; blue boxes were genes not clustered.

To figure out the key genes related to the ruptured plaques, we mapped the 72 hub genes to the DEGs. 45 hub genes with logFC > 1.0 and p-value < 0.05 were screened out. Among them, 12 were upregulated and 33 were downregulated (Table S3). These 45 hub genes were subsequently used to construct the sub-network by Cytoscape 3.7.2 software. The plugin molecular complex detection (MCODE) tool was used to calculate the most significant clusters in the sub-network. As a result shown in Fig. 6 and Table 1, 3 clusters with 13 key genes were figured out. They were LOXL1, FBLN5, FMOD, ELN, EFEMP1 in cluster1, RILP, HLA-DRA, HLA-DMB, HLA-DMA in cluster 2, and SFRP4, FZD6, DKK3 in cluster 3. These genes were considered to be key genes.

**Table 1:** The list of key genes.

	Gene symbol	MCODE score	Official full name
Cluster 1	FMOD	3	Fibromodulin
	EFEMP1	3	EGF containing fibulin extracellular matrix protein 1
	LOXL1	2.7	Lysyl oxidase like 1
	BGN	2.7	Biglycan
	FBLN5	2.4	Fibulin 5
	ELN	2.4	Elastin
Cluster 2	RILP	3.0	Rab interacting lysosomal protein
	HLA-DRA	3.0	Major histocompatibility complex, class II, DR alpha
	HLA-DMB	3.0	Major histocompatibility complex, class II, DM beta
	HLA-DMA	3.0	Major histocompatibility complex, class II, DM alpha
Cluster 3	SFRP4	2.0	Secreted frizzled related protein 4
	FZD6	2.0	Frizzled class receptor 6
	DKK3	2.0	Dickkopf WNT signaling pathway inhibitor 3

### Validation of key genes in GSE120521 and human CEA plaques

**Figure 7:**
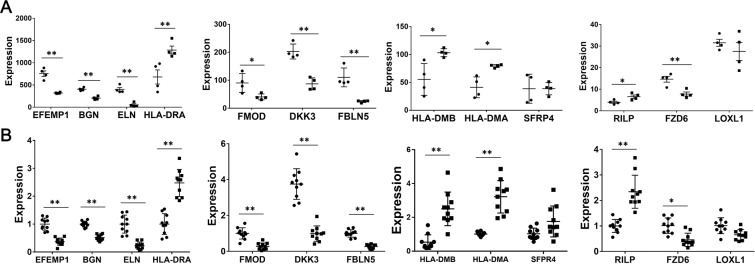
The expression of 13 key genes in GSE120521 and human CEA plaques. (**A**) the expression of 13 key genes in GSE120521. Black round: stable plaques; black square: ruptured plaques; n = 4 for both stable and ruptured plaques. (**B**) the expression of 13 key genes in human CEA plaques. n = 10 for both stable and ruptured plaques. *P < 0.05, **P < 0.01 vs Stable group.

To further validate the key genes, we detected the expression of the 13 key genes in the microarray profile GSE120521, and in the human CEA plaques (10 stable and 10 ruptured). In GSE120521, EFEMP1, BGN, ELN, FMOD, DKK3, FBLN5, and FZD6 were decreased in ruptured plaques, while HLA-DRA, HLA-DMB, HLA-DMA, and RILP were increased. (Fig. 7A). Similar results were found in human CEA plaques (Fig. 7B).

## Discussion

In the present study, six modules were detected by WGCNA using the dataset GSE41571. Among the six modules, blue and brown modules were associated with the rupture of atherosclerotic plaques significantly. 72 hub genes were screened out from the blue and brown modules. After mapping the 72 hub genes to the DEGs, 45 genes were found and used to construct a sub-network. Eventually, 13 key genes were identified. The expression levels of EFEMP1, BGN, ELN, FMOD, DKK3, FBLN5, FZD6, HLA-DRA, HLA-DMB, HLA-DMA, and RILP were significantly changed in ruptured plaques. These genes may play critical roles in the stability of atherosclerotic plaques.

The stability of atherosclerotic plaques is now considered to be essential in the clinical events of atherosclerosis. Studies have shown the abundant presence of inflammatory cells, such as monocyte-derived macrophages and T-lymphocytes at the site of ruptured plaques. The mediators secreted by the inflammatory cells lead to the activation and proliferation of smooth muscle cells, lesion progression and matrix degradation of plaque fibrous cap^13^. Therefore, the active inflammation and matrix degradation might be the two most crucial processes that contribute to the rupture of plaques. However, the molecular mechanisms are still unclear.

Previous studies compared the gene expression profile between unstable plaques and normal tissues^14^. They screened out the DEGs from GSE41571, GSE118481, and E-MTAB-2055, and analyzed the functions and mechanisms of these DEGs by GO and KEGG enrichment. The genes COL1A2, ADCY3, CXCR4, TYROBP, and IGFBP6 were thought to play roles in atherosclerotic plaques^14^. Another research analyzed the DEGs in GSE41571^10^. COL3A1, COL1A2, ASPN, PPBP were thought to probably play important roles in the rupture of atherosclerotic plaques^10^. Our research used the WGCNA to construct the co-expression network. The modules with high association with the rupture of plaques were identified first. And then we mapped the hub genes to DEGs. The key genes screened out by this method were based on not only the expression level, but also the ruptured traits of the plaques.

In the present study, we identified six modules through WGCNA analysis. Among these six modules, blue and brown modules were with the highest correlations with plaque traits (with a correlation of 0.82 and −0.9 respectively); 72 hub genes were identified in these two modules. Further GO enrichment of the 72 hub genes from blue and brown modules indicates these genes be mostly related to cell adhesion, extracellular matrix organization, cell growth, cell migration, and leukocyte migration. These five processes are related to the key pathogenic steps of atherosclerosis progression and plaque rupture. In KEGG enrichment, terms of PI_3_K-Akt signaling, focal adhesion, ECM-receptor interaction were enriched. Focal adhesion, also named cell-matrix adhesion, is a structure which assembles the extracellular matrix (ECM) and interacting cells, through which regulatory signals are transmitted^15^. The interaction between ECM and cells is also mediated by some transmembrane receptors, such as integrins and CD36. The cell adhesion, migration, differentiation, proliferation and apoptosis, and the component of ECM are controlled by the interactions between ECM and cells. PI_3_K-Akt signaling is an important signaling pathway, which is included in variable processes such as protein synthesis, cell survival, apoptosis, proliferation, metabolism, etc. Taking all these together, the hub genes may affect the signal transduction and subsequent cell adhesion, migration, proliferation, and ECM degradation, which eventually lead to the progress and rupture of plaques.

Further analysis discovered 13 key genes. Among them, EFEMP1, BGN, ELN, FMOD, DKK3, FBLN5, and FZD6 were decreased in ruptured plaques, while HLA-DRA, HLA-DMB, HLA-DMA, and RILP were downregulated.

HLA-DRA, HLA-DMB, and HLA-DMA belong to the major histocompatibility complex, class II. They participate in the adaptive immune response and T cell receptor signaling pathway. They are binding peptides derived from antigens that access the endocytic route of antigen presenting cells (APC) and presents them on the cell surface for recognition by the CD4 T-cells^16^. The site of intimal rupture or erosion of thrombosed coronary atherosclerotic plaques were always characterized by abundant expression of HLA antigens on both inflammatory cells and adjacent smooth muscle cells, suggesting an active inflammatory reaction^17^. Our present research identified HLA-DRA, HLA-DMB, and HLA-DMA are key genes for the rupture of the plaques. The mechanisms may relate to the inflammation in atherosclerotic plaques. RILP encodes Rab interacting lysosomal protein. It is involved in the regulation of lysosomal morphology and distribution^18^. It promotes the centripetal migration of phagosomes and the fusion of phagosomes with the late endosomes and lysosomes^18^. However the mechanisms of RILP on atherosclerosis are still unknown. Based on the results of our present study, RILP might be a new target for the pathogenesis of atherosclerosis. BGN encodes biglycan. It is involved in collagen fiber assembly^19^. FMOD encodes fibromodulin. It affects the rate of fibrils formation and has a primary role in collagen fibrillogenesis^20^. FBLN5 encodes fibulin 5, while ELN encodes elastin. Elastin is a major structural protein of aorta^21^. It controls the stabilization of arterial structure by regulating proliferation and organization of vascular smooth muscle^22^. FBLN5 is essential for elastic fiber formation. It is involved in the assembly of continuous elastin polymer and promotes the interaction of microfibrils and ELN^21^. These genes might contribute to the pathogenesis of atherosclerosis via affecting the collagen and the fibrous cap stability. FZD6 encodes the protein frizzled-6, a 7 transmembrane protein receptor for Wnt4^23,24^. FZD6 mediates the functions of Wnt signaling by both canonical and non-canonical pathways, but mainly through non-canonical pathway. The activation of Wnt4/FZD6 involves in the different diseases such as cancer, embryonic development, bone marrow mesenchymal stem cell dysfunction^23,25,26^. EFEMP1 encodes EGF containing fibulin extracellular matrix protein 1. EFEMPL binds to EGFR and play roles in cell adhesion and migration^27^. However, the roles of FZD6 and EFEMP1 in atherosclerosis are still unclear. Our present study indicates FZD6 might be a novel mechanism of atherogenesis. DKK3 encodes protein dickkopf homolog 3, a secreted glycoprotein belongs to the dickkopf family. In the cardiovascular system, DKK3 is involved in the regulation of cardiac remodeling and vascular smooth muscle differentiation^28,29^. In atherosclerotic mice, DKK3 deficiency caused the acceleration of atherosclerosis and led to the vulnerable atherosclerotic plaques due to the reduction of the number of SMCs and matrix protein deposition^23,30^. Further mechanism study on DKK3 is needed.

Our study still has some limitations. According to FAQ’s of WGCNA package, no less than 15 samples are recommended. However, in our case, only 11 samples were analyzed. Therefore, we need to be aware of several problems with small sample size, such as poorer fit to scale-free topology, and the resulted network may be too noisy to be biologically meaningful. And another limitation is the lack of clinical trait data in the involved GEO datasets. These may affect the precision of present results on certain subgroup of patients.

Overall, in our study, we used the WGCNA to identify the key genes for the ruptured traits of atherosclerotic plaques. 13 key genes were screened out. These genes may play crucial roles in inflammation, fibrous cap formation and degradation, and lead to the rupture of atherosclerotic plaques.

## Materials and Methods

### Data collection and preprocessing

The gene expression profiles of GSE41571 and GSE120521 were downloaded from the Gene Expression Omnibus (GEO, https://www.ncbi.nlm.nih.gov/geo/). The GSE41571 was a genome-wide expression profile of macrophage-rich regions of 5 ruptured and 6 stable human atherosclerotic plaques. They were obtained from carotid endarterectomy operation. The clinical, radiological and histopathological criteria were used to evaluate the plaques as stale or ruptured. Laser micro-dissection was used to obtain the macrophage-rich regions. After amplification of total RNA of these samples, the transcriptional profiling was performed using GPL570 platform. The GSE120521 was RNA-seq profile of 4 stable and 4 ruptured human atherosclerotic plaques. The platform of GPL16791 Illumina HiSeq. 2500 was used.

The preprocessing of the two series matrix profiles was conducted on R statistical software version 3.4.2 with related packages from Bioconductor (www.bioconductor.org). After background correction, the original data were quantile normalized using Robust Multi-array Average (RMA) algorithm in the affy package in Bioconductor.

### DEGs identification

The two series matrix files were annotated with official gene symbols using the platform file and annotation package in the R software. The “LIMMA” package in the R was used to identify the DEGs. Genes with a logFC > 1.0 and *p*-value < 0.05 were considered to be DEGs. The heatmap of these DEGs were conducted using software “MeV”. “ggplot2” package in R software was used to produce the volcano plot. All the packages used in R software were downloaded from Bioconductor (http://www.bioconductor.org).

### Co-expressed module identification by WGCNA

The WGCNA method was used to construct the co-expression network and to identify the co-expressed modules. The WGCNA was conducted in the R software using WGCNA package. The GSE41571 dataset includes 8413 genes. Among them, the top 50% most variantly expressed genes (4207 genes) were used for further WGNCA analysis. The WGCNA was conducted as previously reported^31^. Briefly, the similarity matrix S_*ij*_ was constructed by calculating the Pearson’s correlation coefficient between each pair of genes *i* and *j*. The adjacency matrix A_*ij*_ was obtained by calculating the connection strength between gene *i* and *j*. The formulas were as follows:

$${({S}_{ij})}^{signed}=|(1+{\rm{cor}}({X}_{i},{Y}_{j}))/2|$$

$${A}_{ij}=power({S}_{ij},\beta )$$

Where *Xi* and *Yj* were vectors of expression values and S*ij* was the Pearson’s correlation coefficient of gene *i* and *j*; A_*ij*_ represented the network strength between gene *i* and *j*. A power of β = 9 was selected in the present study as the soft-thresholding parameter to ensure a scale-free network.

To identify the co-expressed gene modules, the adjacency metrix was changed to topological matrix by the method of TOM. The formula for TOM was as follows:

$$TO{M}_{ij}=\frac{{\sum }_{K=1}^{N}{A}_{ik}\cdot {A}_{kj}+{A}_{ij}}{\min ({K}_{i},{K}_{j})+1-{A}_{ij}}$$

With the minimum gene group size set as 20 for the gene dendrogram, the TOM-based dissimilarity measure was adopted to perform the average linkage hierarchical clustering. Using the Dynamic Tree Cut algorithm, genes were categorized into the same gene modules if they had similar expression profiles.

### Relations between identified modules and plaque straits

To identify the gene modules which were highly related to the ruptured traits of atherosclerotic plaques, module eigengenes (MEs) were firstly identified. MEs were principal components of a gene module. The expression of MEs represented all genes in the given module. The clinically significant modules were identified by calculating the correlation between MEs and ruptured traits of plaques. The gene significance (GS) and module significance (MS) were also calculated to identify the relations between gene expression and ruptured traits of plaques. Once the linear regression model of ruptured traits of plaques vs. gene expression was obtained, the GS was then defined as the mediated *P*-value of each gene (GS = lg*P*) in the model. The MS was defined as the average GS of all the genes involved in the module. MS was measured to incorporate clinical information into the coexpression network.

### Hub genes identification

There is a tendency that, in a given module, the hub genes have strong correlations with certain clinical traits. To identify the hub genes of a module, the correlation between ruptured traits of plaques and gene expression were measured by the absolute value of Pearson’s correlation (cor.gene TraitSignificance>0.2). The inter-module connectivity of genes was also measured by the absolute value of the Pearson’s correlation. Genes with cor.geneModuleMembership>0.9 were considered to be hub genes.

### Gene ontology and pathway enrichment analysis

The DAVID Functional Annotation Bioinformatics Microarray Analysis (website:https://david.ncifcrf.gov/) was used for the GO enrichment and KEGG pathway analysis. The GO enriched the genes into 3 terms: Molecular Function (MF), Biological Process (BP), and Cellular Component (CC). A *q*-value<0.05 was considered to be statistically significant for the correlations. Through GO and KEGG enrichment, the possible functions or biological processes of the genes in a given module were predicted. The Bubble Charts were conducted using the OmicShare tools, a free online platform for data analysis (http://www.omicshare.com/tools). The RichFactor was calculated by “counts”/“pop hits”. The “counts” is the number of hub genes enriched in a certain term. The “pop hits” is the number of all genes enriched in a certain term.

### Protein-protein interaction network integration

The STRING online tool (website: https://string-db.org/) was used for the identification of protein-protein interactions. The STRING database covers the number of 9 643 763 proteins from 2 031 organisms. It provides direct (physical) interactions and indirect (functional) associations; they stem from computational prediction, from knowledge transfer between organisms, and from interactions aggregated from other (primary) databases. The main databases used for STRING are the Genomic Context Predictions, the High-throughput Lab Experiments, the (Conserved) Co-Expression, the Automated Textming, and the Previous Knowledge in Databases.

### Key gene identification and validation

To figure out the key genes, we firstly mapped the hub genes to the DEGs and obtained the logFC and p-value of each hub gene. Then we screened out the hub genes with logFC>1.0 and p-value <0.05. These genes were used to construct the sub-network using Cytoscape 3.7.2 software. The plugin “MCODE” tool in the “Apps” of the Cytoscarpe was used to calculate the most significant clusters of genes. The genes in the clusters were considered to be key genes.

To validate the key genes, the expression profile of dataset GSE120521 was downloaded from GEO, and the key genes expression were analyzed.

For further validation, human CEA plaques were used. A total of 10 patients with stable plaques and 10 patients with ruptured plaques were involved. All patients were performed CEA from the First Hospital of Jilin University (Changchun, Jilin, China) from July, 2019 to November, 2019. The procedures were approved by the Ethics Committee of the First Hospital of Jilin University (No.2019-272, Changchun, Jilin). Written informed consent was obtained from every participant.

The surgical specimens were obtained and fast-frozened in liquid nitrogen, then they were stored at −80 °C until use. The evaluation of stable and unstable plaques was conducted according to the classification defined by the American Heart Association (AHA) and previous report^32^. The type I/II plaques are with near-normal wall thickness but no calcification; the type III plaques are small eccentric plaque, or plaque with diffuse intimal thickening, but no calcification; the type IV/V plaques has lipid or necrotic core, which is surrounded by fibrous tissue, and with possible calcification; the type VI are complex plaque with possible surface defect, haemorrhage or thrombus; the type VII are plaques with obvious calcification; the type VIII are fibrotic plaque with possible small calcification, but without lipid core^32^. In the present study, the type I-II, III, VII, VIII were considered stable plaques, while type IV-V, VI to be ruptured plaques. The classification of carotid artery was performed by two independent investigators.

The expressions of key genes in these plaques were analyzed using qPCR as previously reported by our lab^33^. Briefly, the total RNA was extracted by Trizol. After reverse transcripted to cDNA, the qPCR reaction was conducted using the SYBR Green Premix DimerEraser Kit (TaKaRa Bio Inc., Dalian, China) on ABI QuantaStudio5 system. The qPCR program was: 95 °C for 30 s, followed by 40 cycles of 95 °C 5 s, 60 °C 30 s. The primers used were listed in Table S3. All samples were run in triplicate. The results were calculated by the 2^(−ΔΔCq)^ Method^34^.

## Supplemenatry information

Supplemenatry information.
Supplemenatry information2.
Supplemenatry information3.
