# Supplementary material for: Identification of key genes in ruptured atherosclerotic plaques by weighted gene correlation network analysis
Source: Sci Rep. 2020 Jul 2;10:10847. doi: 10.1038/s41598-020-67114-2 (PMC7331608; doi:10.1038/s41598-020-67114-2)
Supplement: Supplementary file 1 — Supplemenatry information. [file 41598_2020_67114_MOESM1_ESM.docx]

**Identification of key genes in ruptured atherosclerotic plaques by weighted gene correlation network analysis**

Bao-Feng Xu^1*^, Rui Liu^2*^, Chun-Xia Huang^3,4^, Bin-Sheng He^4^, Guang-Yi Li^4^, Hong-Shuo Sun^5,6^, Zhong-Ping Feng^5*^, Mei-Hua Bao^3,4#^

^1^ First Hospital of Jilin University, Changchun, Jilin, 130021, China

^2^ Department of VIP Unit, China-Japan Union Hospital of Jilin University, Changchun 130033, China

^3^ Science Research Center, Changsha Medical University, Changsha, 410219, China

^4^ Academician Workstation, Changsha Medical University, Changsha, 410219, China

^5^ Department of Surgery, Faculty of Medicine, University of Toronto, Toronto, ON, Canada

^6^ Department of Physiology, Faculty of Medicine, University of Toronto, Toronto, ON, Canada

^*^ These two authors contribute equally to this work

Author contributions: Xu B.-F.collected the human specimens; Liu R., Huang C.-X., and Li G.-Y. performed the experiments; Bao M.-H. and Feng Z.-P. designed the experiments; Bao M.-H. wrote the main manuscript text; He B.-S. and Sun H.-S. revised the manuscript. All authors reviewed the manuscript.

^#^ Authors to whom correspondence should be addressed:

**Correspondence:**

**Zhong-Ping Feng, MD, MSc, PhD**

Department of Physiology

Faculty of Medicine, University of Toronto, 1 King's College Circle

Toronto, Ontario, Canada  M5S 1A8

Email: zp[.feng@utoronto.ca](mailto:.feng@utoronto.ca)

Phone: +1 416 946-0671

**Mei-Hua Bao, Ph.D**

Science Research Center, Changsha Medical University, Changsha, 410219, China

Email: mhbao78@163.com;

Tel: +86 731 88602602; Fax: +86 731 88602602

**Keywords:** Atherosclerosis, ruptured plaque, weighted gene correlation network analysis, biomarker

**Running title:** XU et al: WGCNA IDENTIFICATION OF KEY GENES FOR RUPTURED PLAQUES

**Table S1 The top 40 upregulated and top 40 downregulated genes in GSE41571**

| **row.names(tT)** | **logFC** | **AveExpr** | **P.Value** |
| --- | --- | --- | --- |
| COL21A1 | -5.20744 | -0.70814 | 3.16E-05 |
| FMO2 | -4.69016 | 0.71392 | 0.000115 |
| FNDC1 | -4.52824 | -0.02283 | 3.42E-05 |
| CRISPLD1 | -4.50849 | 0.753911 | 0.000796 |
| ITGBL1 | -4.26299 | -0.78566 | 2.07E-05 |
| SFRP2 | -4.22537 | -0.18651 | 0.001131 |
| SFRP4 | -4.09638 | -0.09828 | 0.001913 |
| KCNT2 | -4.01468 | 0.523086 | 0.00013 |
| HMCN1 | -4.00993 | -0.31447 | 0.000171 |
| OMD | -3.87718 | 0.861459 | 0.001554 |
| ACTA2 | -3.85592 | 1.10302 | 0.000312 |
| AK021804 | -3.78151 | 0.183532 | 0.000545 |
| MIR100HG | -3.73139 | -0.35825 | 0.000506 |
| FAT4 | -3.72973 | 0.065598 | 0.000135 |
| OGN | -3.69964 | 0.385209 | 0.007489 |
| ZFPM2 | -3.68466 | -0.16348 | 6.00E-05 |
| MGP | -3.62909 | 0.466968 | 0.000411 |
| MYH10 | -3.57566 | -0.46437 | 0.000638 |
| AOC3 | -3.54197 | -0.33376 | 0.001136 |
| NTN4 | -3.51571 | -0.28486 | 0.000265 |
| SGIP1 | -3.51457 | -0.74095 | 0.000152 |
| COMP | -3.38371 | 0.131622 | 0.017045 |
| PRELP | -3.36157 | -0.61392 | 0.000865 |
| LTBP1 | -3.33914 | -0.64998 | 0.000223 |
| ISLR | -3.31409 | -0.12428 | 0.000269 |
| OLFML1 | -3.30549 | -0.09208 | 0.001035 |
| ITGA10 | -3.23983 | -0.11384 | 0.0003 |
| PLSCR4 | -3.22006 | -0.09149 | 4.27E-05 |
| GUCY1A3 | -3.2132 | 0.155438 | 0.002222 |
| NEXN | -3.18151 | -0.41898 | 0.000635 |
| PODXL | -3.13363 | 0.431994 | 0.00101 |
| NAP1L3 | -3.06512 | -0.64296 | 4.56E-05 |
| RCAN2 | -3.02856 | 0.035212 | 0.013938 |
| LOC728061 | -3.02825 | -0.29454 | 0.001917 |
| AEBP1 | -3.02758 | -0.56314 | 0.001312 |
| DNM3OS | -3.01459 | 0.014822 | 0.000445 |
| FAP | -3.01456 | -1.02056 | 3.32E-05 |
| HECW2 | -2.99028 | -0.06078 | 4.70E-05 |
| PCDHB16 | -2.9898 | 0.349366 | 0.003117 |
| TMEM47 | -2.98898 | -0.0262 | 0.000814 |
| C1QA | 2.032552 | -0.56815 | 0.00623 |
| CKAP2 | 2.036331 | 0.03031 | 0.008557 |
| IGLC1 | 2.048809 | 0.387009 | 0.01386 |
| CTSLP8 | 2.084271 | 0.076614 | 0.000427 |
| CKAP2 | 2.137245 | 0.306625 | 0.004879 |
| CD52 | 2.151269 | 0.382173 | 0.004824 |
| IGLJ3 | 2.20477 | -0.00904 | 0.0023 |
| abParts | 2.223917 | 0.453489 | 0.006365 |
| PLD3 | 2.227606 | -0.78633 | 0.002127 |
| MS4A6E | 2.233247 | 0.20183 | 0.001782 |
| S100A9 | 2.315078 | -0.40235 | 0.000482 |
| CKAP2 | 2.37221 | -0.03945 | 0.002975 |
| LINC00847 | 2.373563 | 0.11533 | 0.000356 |
| DHRS9 | 2.381444 | 0.171952 | 0.000632 |
| CD72 | 2.427997 | -0.27701 | 0.002718 |
| IGLL3P | 2.576982 | -0.05965 | 0.003761 |
| IGKV1-37 | 2.581744 | -0.27321 | 0.006139 |
| HMOX1 | 2.590269 | -0.35708 | 0.00102 |
| TNFRSF17 | 2.606314 | 0.738404 | 0.007114 |
| LEP | 2.724368 | 0.336076 | 0.000527 |
| CKAP2 | 2.758082 | -0.08148 | 0.00636 |
| CD38 | 2.781624 | 0.765909 | 1.01E-06 |
| IGKV1OR2-108 | 2.824994 | 0.210823 | 0.001382 |
| FABP4 | 2.831332 | 0.217237 | 0.000543 |
| IGK | 2.849579 | -0.50529 | 0.009133 |
| FCGR3A | 2.916393 | -0.69764 | 0.000467 |
| HLA-DQB1 | 2.929387 | -1.24879 | 0.04783 |
| IGH | 3.164691 | -1.26976 | 0.026744 |
| IGLJ3 | 3.20131 | 0.814251 | 0.011122 |
| IGLL5 | 3.208723 | 0.703558 | 0.006905 |
| METTL7B | 3.337815 | -0.1519 | 0.0035 |
| IGKC | 3.380923 | 0.249341 | 0.008027 |
| HBA1 | 3.434246 | -0.6697 | 0.007425 |
| IGJ | 3.531607 | -0.88963 | 0.033452 |
| HBB | 3.5974 | -0.75423 | 0.011377 |
| MZB1 | 3.646071 | 0.557025 | 0.001105 |
| IGKV1-17 | 3.718865 | 0.129682 | 0.002899 |
| IGHG1 | 3.985523 | -1.09963 | 0.022343 |
| PPBP | 4.084296 | 1.715036 | 0.00069 |
| IGLC1 | 4.192301 | -0.08411 | 0.002573 |
