# Supplementary material for: Identification of key genes in ruptured atherosclerotic plaques by weighted gene correlation network analysis
Source: Sci Rep. 2020 Jul 2;10:10847. doi: 10.1038/s41598-020-67114-2 (PMC7331608; doi:10.1038/s41598-020-67114-2)
Supplement: Supplementary file 2 — Supplemenatry information2. [file 41598_2020_67114_MOESM2_ESM.docx]

**Identification of key genes in ruptured atherosclerotic plaques by weighted gene correlation network analysis**

Bao-Feng Xu^1*^, Rui Liu^2*^, Chun-Xia Huang^3,4^, Bin-Sheng He^4^, Guang-Yi Li^4^, Hong-Shuo Sun^5,6^, Zhong-Ping Feng^5*^, Mei-Hua Bao^3,4#^

^1^ First Hospital of Jilin University, Changchun, Jilin, 130021, China

^2^ Department of VIP Unit, China-Japan Union Hospital of Jilin University, Changchun 130033, China

^3^ Science Research Center, Changsha Medical University, Changsha, 410219, China

^4^ Academician Workstation, Changsha Medical University, Changsha, 410219, China

^5^ Department of Surgery, Faculty of Medicine, University of Toronto, Toronto, ON, Canada

^6^ Department of Physiology, Faculty of Medicine, University of Toronto, Toronto, ON, Canada

^*^ These two authors contribute equally to this work

Author contributions: Xu B.-F.collected the human specimens; Liu R., Huang C.-X., and Li G.-Y. performed the experiments; Bao M.-H. and Feng Z.-P. designed the experiments; Bao M.-H. wrote the main manuscript text; He B.-S. and Sun H.-S. revised the manuscript. All authors reviewed the manuscript.

^#^ Authors to whom correspondence should be addressed:

**Correspondence:**

**Zhong-Ping Feng, MD, MSc, PhD**

Department of Physiology

Faculty of Medicine, University of Toronto, 1 King's College Circle

Toronto, Ontario, Canada  M5S 1A8

Email: zp[.feng@utoronto.ca](mailto:.feng@utoronto.ca)

Phone: +1 416 946-0671

**Mei-Hua Bao, Ph.D**

Science Research Center, Changsha Medical University, Changsha, 410219, China

Email: mhbao78@163.com;

Tel: +86 731 88602602; Fax: +86 731 88602602

**Keywords:** Atherosclerosis, ruptured plaque, weighted gene correlation network analysis, biomarker

**Running title:** XU et al: WGCNA IDENTIFICATION OF KEY GENES FOR RUPTURED PLAQUES

**Table S2 The hub genes identified by WGCNA Hub genes**

| Merged  Colors | Freq | Hub genes No. | Names |
| --- | --- | --- | --- |
| Blue | 319 | 48 | SVIL, FOXC1, AEBP1, GULP1, PCOLCE, DKK3, PRELP, AMOTL2, GSTA4, PTPRK, BGN, RCAN2, FRY, PTPN13, AJUBA, CDC42BPA, FMOD, FGF7, PAWR, PKIG, LOXL1, TRIM2, NOV, FZD6, TJP2, FAM13C, LOC101928916, KIAA0355, PPP1R3C, CAV2, SFRP4, C8orf88, NET1, LDOC1, MID1, EFEMP1, COL16A1, TJP1, HEPH, TPBG, IGFBP7, SCG2, FBLN5, GEM, GLB1, RAB7B, CD53, SLC2A5 |
| Brown | 124 | 24 | TIMP2, FUCA1, TMEM37, HLA-DMB, S100A9, CTSC, MGAT1, SLCO2B1, AKR1B1, B3GNT7, SCCPDH, QPRT, C2, LGMN, CTSA, BLVRB, ASAH1, SCARB1, MS4A4A, CD300LF, ITPRIPL2, HDGFRP3, SRSF11, C8orf59 |
| Green | 51 | 15 | TOP2A, ENTHD1, SLC16A1, AHNAK2, ADAM12, ZNF638, LINC00622, EZH2, SLC9B2, DLGAP5, SEC61G, MSANTD3, CD302, SMARCD3, SERPING1 |
| Red | 22 | 14 | RPL18, RPS5, MIR4691, MIR6805, COX6A1, RPL29, COX5B, SNRPD2 , TUBG1, CST3,LIMK2, TSC22D2, MIR4680, FAM178A, |
| Turquoise | 391 | 103 | LUZP1, YTHDC2, MED1, TM2D1, EXOSC1, MAP4K5, ERCC5, CLIP1, MSH6, FN1, KRR1, ZKSCAN1, SMCHD1, TTC30B, FAM126B, PNN, CEP57, TAF11, VAPB, RSRC2, CDC42BPB, CHD4, FCHO2, SRRM1, CWC27, SF3B1, FKBP15, DCBLD1, KDM4C, EIF3A, RNF141, HNRNPDL, PPP2R5E, TRIM52, VTI1A, ITGB1, THOC1, HNRNPH1, BCLAF1, SON, HSP90B1, MTIF2, WBP4, SSB, ITGAV, ZAK, RBM25, SRSF10, PSD3, TTC3, GON4L, TACC1, FXR1, SPEN, HSPA9, PSMA4, STRAP, CBR4, JPX, HMGB1, GNL2, SPPL3, NPEPPS, GPATCH11, RP11-690I21.2, TCERG1, STXBP3, PDXDC1, PRKAB2, SLC5A3, EPM2AIP1, CTAGE5, MAP4K4, ZC3H15, EPRS, TRIM4, LOC100131541, ZBTB43, SERPINB6, BD495725, HNRNPH2, C5orf28, SEC63, AX747652, PEPD, HAX1, LINC00847, TAF10, LGALS1, GPX4, COPE, IL12RB1, NAGLU, GYPC, GPAA1, CAPNS1, INTS9, TMEM147, DNPH1, CIB1, DAP, GSTP1, ACOT8 |
| Yellow | 88 | 32 | PLD3, STK10, CTSD, PCSK7, SNX17, ASNA1, GNB2, P4HB, PLA2G15, CYBA, OAZ2, MLF2, PSMD3, KIAA0195, APEH, SLC3A2, LYPLA2, TPD52L2, PPP1CA, RHOG, EIF4A1, GAA, ILVBL, HCLS1, BCAP31, BCKDK, CD81, GRN, LRRC41, SYNGR2, NINJ1, ACP2 |
