# Supplementary material for: Identification of key genes in ruptured atherosclerotic plaques by weighted gene correlation network analysis
Source: Sci Rep. 2020 Jul 2;10:10847. doi: 10.1038/s41598-020-67114-2 (PMC7331608; doi:10.1038/s41598-020-67114-2)
Supplement: Supplementary file 3 — Supplemenatry information3. [file 41598_2020_67114_MOESM3_ESM.docx]

**Identification of key genes in ruptured atherosclerotic plaques by weighted gene correlation network analysis**

Bao-Feng Xu^1*^, Rui Liu^2*^, Chun-Xia Huang^3,4^, Bin-Sheng He^4^, Guang-Yi Li^4^, Hong-Shuo Sun^5,6^, Zhong-Ping Feng^5*^, Mei-Hua Bao^3,4#^

^1^ First Hospital of Jilin University, Changchun, Jilin, 130021, China

^2^ Department of VIP Unit, China-Japan Union Hospital of Jilin University, Changchun 130033, China

^3^ Science Research Center, Changsha Medical University, Changsha, 410219, China

^4^ Academician Workstation, Changsha Medical University, Changsha, 410219, China

^5^ Department of Surgery, Faculty of Medicine, University of Toronto, Toronto, ON, Canada

^6^ Department of Physiology, Faculty of Medicine, University of Toronto, Toronto, ON, Canada

^*^ These two authors contribute equally to this work

Author contributions: Xu B.-F.collected the human specimens; Liu R., Huang C.-X., and Li G.-Y. performed the experiments; Bao M.-H. and Feng Z.-P. designed the experiments; Bao M.-H. wrote the main manuscript text; He B.-S. and Sun H.-S. revised the manuscript. All authors reviewed the manuscript.

^#^ Authors to whom correspondence should be addressed:

**Correspondence:**

**Zhong-Ping Feng, MD, MSc, PhD**

Department of Physiology

Faculty of Medicine, University of Toronto, 1 King's College Circle

Toronto, Ontario, Canada  M5S 1A8

Email: zp[.feng@utoronto.ca](mailto:.feng@utoronto.ca)

Phone: +1 416 946-0671

**Mei-Hua Bao, Ph.D**

Science Research Center, Changsha Medical University, Changsha, 410219, China

Email: mhbao78@163.com;

Tel: +86 731 88602602; Fax: +86 731 88602602

**Keywords:** Atherosclerosis, ruptured plaque, weighted gene correlation network analysis, biomarker

**Running title:** XU et al: WGCNA IDENTIFICATION OF KEY GENES FOR RUPTURED PLAQUES

**Table S3 45 genes used for construction of sub-network**

| **gene** | **Relation** | **P.value of correlation** | **logFC** | **AveExpr** | **P.Value of expression** |
| --- | --- | --- | --- | --- | --- |
| S100A9 | 0.967231 | 1.17E-06 | 2.315078 | -0.40235 | 0.000482 |
| SLCO2B1 | 0.94989 | 7.68E-06 | 1.78899 | -0.31122 | 0.001295 |
| MS4A4A | 0.90595 | 0.000122 | 1.440632 | -0.32169 | 0.028572 |
| CTSA | 0.931849 | 2.99E-05 | 1.402146 | -0.40984 | 0.001527 |
| C2 | 0.93557 | 2.33E-05 | 1.401546 | -0.54348 | 0.007136 |
| CD300LF | 0.903236 | 0.000139 | 1.378085 | -0.38871 | 0.031245 |
| HLA-DMB | 0.968243 | 1.01E-06 | 1.210963 | -0.37715 | 0.008894 |
| AKR1B1 | 0.946423 | 1.03E-05 | 1.206646 | -0.49353 | 0.004324 |
| RAB7B | -0.91712 | 7.05E-05 | 1.11533 | -0.18583 | 0.007253 |
| BLVRB | 0.929514 | 3.46E-05 | 1.083904 | -0.14662 | 0.004276 |
| MGAT1 | 0.951678 | 6.54E-06 | 1.058399 | -0.41517 | 0.001986 |
| LGMN | 0.935382 | 2.36E-05 | 1.049247 | -0.3728 | 0.008765 |
| KIAA0355 | 0.925594 | 4.39E-05 | -1.01261 | 0.067169 | 0.010674 |
| AJUBA | 0.943371 | 1.32E-05 | -1.02719 | 0.302748 | 0.033413 |
| HDGFRP3 | -0.91125 | 9.51E-05 | -1.08707 | 0.03875 | 2.00E-05 |
| MID1 | 0.919317 | 6.27E-05 | -1.20801 | 0.218105 | 0.008761 |
| TPBG | 0.906008 | 0.000122 | -1.30721 | -0.04104 | 0.00023 |
| PTPN13 | 0.941498 | 1.52E-05 | -1.33554 | 0.016243 | 0.005031 |
| PPP1R3C | 0.924577 | 4.66E-05 | -1.33724 | 0.430965 | 0.057665 |
| LOC101928916 | 0.925645 | 4.38E-05 | -1.36069 | -0.07812 | 0.010584 |
| PTPRK | 0.951169 | 6.85E-06 | -1.37303 | 0.132008 | 0.003669 |
| GEM | 0.900719 | 0.000155 | -1.4077 | -0.24483 | 0.027647 |
| FAM13C | 0.92753 | 3.91E-05 | -1.43875 | 0.46656 | 0.0194 |
| IGFBP7 | 0.904703 | 0.00013 | -1.45609 | -0.14309 | 0.001735 |
| FBLN5 | 0.902295 | 0.000145 | -1.50567 | 0.188699 | 0.014917 |
| NET1 | 0.920866 | 5.76E-05 | -1.59651 | 0.250305 | 0.00462 |
| LDOC1 | 0.91995 | 6.05E-05 | -1.64045 | 0.689459 | 0.017545 |
| PKIG | 0.932473 | 2.87E-05 | -1.68306 | 0.131474 | 0.006216 |
| GSTA4 | 0.951389 | 6.72E-06 | -1.82912 | 0.408892 | 0.001191 |
| CAV2 | 0.923606 | 4.93E-05 | -1.88139 | -0.24964 | 0.004691 |
| LOXL1 | 0.932185 | 2.92E-05 | -2.12032 | 0.135333 | 0.006588 |
| EFEMP1 | 0.91377 | 8.38E-05 | -2.16961 | -0.06866 | 0.003902 |
| NOV | 0.928341 | 3.72E-05 | -2.36879 | 0.121396 | 0.006569 |
| COL16A1 | 0.909171 | 0.000105 | -2.38581 | 0.233631 | 0.002557 |
| DKK3 | 0.954403 | 5.06E-06 | -2.48796 | -0.45603 | 0.002799 |
| FMOD | 0.93551 | 2.34E-05 | -2.51684 | -0.10919 | 0.004383 |
| FOXC1 | 0.964419 | 1.68E-06 | -2.63652 | 0.170822 | 0.001284 |
| BGN | 0.94857 | 8.62E-06 | -2.70398 | -0.46094 | 0.001695 |
| AMOTL2 | 0.953367 | 5.59E-06 | -2.83743 | 0.054757 | 0.001932 |
| SCG2 | 0.902884 | 0.000141 | -2.88223 | 1.152478 | 0.039526 |
| FZD6 | 0.928254 | 3.74E-05 | -2.95604 | -0.84074 | 0.000607 |
| AEBP1 | 0.964061 | 1.76E-06 | -3.02758 | -0.56314 | 0.001312 |
| RCAN2 | 0.946823 | 9.99E-06 | -3.02856 | 0.035212 | 0.013938 |
| PRELP | 0.953728 | 5.40E-06 | -3.36157 | -0.61392 | 0.000865 |
| SFRP4 | 0.923155 | 5.06E-05 | -4.09638 | -0.09828 | 0.001913 |
